# Draft Genome Sequence of *Kitasatospora cheerisanensis* KCTC 2395, Which Produces Plecomacrolide against Phytopathogenic Fungi

**DOI:** 10.1128/genomeA.00604-14

**Published:** 2014-06-19

**Authors:** Jae Yoon Hwang, Soo Hee Kim, Hye Ryeung Oh, Yong-Joon Cho, Jongsik Chun, Young Ryun Chung, Doo Hyun Nam

**Affiliations:** a College of Pharmacy, Yeungnam University, Gyongsan, South Korea; b Chunlab, Inc., Seoul National University, Seoul, South Korea; c Department of Microbiology, Gyeongsang National University, Jinju, South Korea

## Abstract

*Kitasatospora cheerisanensis* KCTC 2395, which produces antifungal metabolites with bafilomycin derivatives, including bafilomycin C1-amide, was isolated from a soil sample at Mt. Jiri, South Korea. Here, we report its draft genome sequence, which contains 8.04 Mb with 73.6% G+C content and 7,810 protein-coding genes.

## GENOME ANNOUNCEMENT

The genus *Kitasatospora* for actinomycete strains was first suggested by Omura et al. (1). Members of this genus possess different contents of *meso*-diaminopimelic acid and galactose in whole-cell lysate from the closely related members of the *Streptomyces* genus. After a period of debate, the genus *Kitasatospora* was revived by Zhang et al. (2), based on the distinct phylogenetic clades in the 16S rRNA genes as well as a 16S-23S rRNA gene spacer. More recently, the gene for the RNA polymerase β subunit, in addition to the 16S rRNA gene sequence, was employed for the phylogenetic classification of *Kitasatospora* and *Streptomyces* (3). The first genome sequence of *Kitasatospora* was reported by Ichikawa et al. (4), who determined the complete genome sequence of *Kitasatospora setae* NBRC 14216, which produces bafilomycin B1 (setamycin), belonging to the plecomacrolide group.

*K. cheerisanensis* YC75 (KCTC 2395) was isolated from a soil sample at Cheeri-San (Mt. Jiri) in the process of screening biological control agents for a phytopathogenic fungus (5). Later, the antifungal metabolites produced by this strain were confirmed as bafilomycin derivatives, including bafilomycin C1-amide (6).

The genome sequence of *K. cheerisanensis* was obtained with a combination of an Illumina GAIIx 100-bp paired-end library (971.18× coverage), a Roche 454 Titanium 8-kb paired-end library (9.12× coverage), a PacBio 5-kb library (29.68× coverage), and a PacBio 10-kb library (84.06× coverage). The procedure for library construction was performed according to the manufacturers' instructions.

Illumina and PacBio sequencing data were assembled with CLC Genomic Workbench 6.5 (CLCbio, Denmark) and PacBio SMRT Analysis 2.0 using the HGAP2 protocol (Pacific Biosciences, USA). Resulting contigs were scaffolded using GS Assembler 2.6 (Roche Diagnostics, CT). The final assembly provided a total of 5 scaffolds containing 178 contigs. The draft genome of *K. cheerisanensis* consists of 8,035,179 bp, with a 73.6% G+C content. A total of 7,810 coding sequences (CDSs) with 9 rRNA operons and 72 tRNA genes were predicted.

The CDSs were predicted using Glimmer 3.02 (7), and tRNA and rRNA were searched using tRNAscan-SE and HMMER with ezTaxon-e database bacterial rRNA profiles (8–10). The annotation of each CDS was made by homology search against NCBI reference sequence (RefSeq), Clusters of Orthologous Groups (COG), SEED, CatFam, SMART 6.2, PRINTS 42.0, TIGRFAM 13.0, Pfam 27.0, and InterPro 44.0 databases (11–18).

Several gene clusters for the biosynthesis of secondary metabolites were found in the genome, including type I polyketide synthase (PKS) gene clusters (KCH_04080 to KCH_04120 for the biosynthesis of the bafilomycin backbone and KCH_45030 to KCH_45040), a type II PKS gene cluster (KCH_73510 to KCH_73550), nonribosomal peptide synthetase (NRPS) gene clusters (KCH_06280 to KCH_06390, KCH_45400 to KCH_45410, KCH_61460 to KCH_61470, and KCH_70790 to KCH_70800), and PKS/NRPS hybrid gene clusters (KCH_67350 to KCH_67370 and KCH_74020 to KCH_74040).

Genes for resistance to β-lactam antibiotics, including AmpC β-lactamase (KCH_10470 and KCH_10480) and metallo-β-lactamase (KCH_19220 and KCH_36670), were identified. The other putative resistance genes for aminoglycoside antibiotics (KCH_00980 and KCH_00990) and chloramphenicol (KCH_72240 and KCH_73860) were also found in this draft genome.

### Nucleotide sequence accession numbers.

This whole-genome shotgun project has been deposited at DDBJ/EMBL/GenBank under the accession number JNBY00000000. The version described in this paper is version JNBY01000000.
